# Toxic Epidermal Necrolysis (TEN)/Stevens-Johnson Syndrome (SJS) Epidemiology and Mortality Rate at King Fahad Specialist Hospital (KFSH) in Qassim Region of Saudi Arabia: A Retrospective Study

**DOI:** 10.1155/2020/7524726

**Published:** 2020-10-09

**Authors:** Abdullah Alajaji, Jagannath Chandra Shekaran, Omar Mohammed Aldhabbah, Hajar Abdullah Alhindi, Nouf Salem Almazyad, Ziyad Abdulrahman Aljutayli, Saleh Abaalkhail, Saleh Alfouzan

**Affiliations:** ^1^Qassim University, College of Medicine, Buraydah, Saudi Arabia; ^2^Ministry of Health, KFSH, Dammam, Saudi Arabia

## Abstract

**Background:**

Toxic epidermal necrolysis (TEN) and Stevens-Johnson syndrome (SJS) are life-threatening conditions caused by drug reactions. There are multiple causative drugs and different risk factors associated with SJS/TEN.

**Objectives:**

To study the epidemiology of SJS/TEN and associated mortality rate in Qassim region, Saudi Arabia. *Methodology*. A retrospective chart review of all patients with the diagnosis of SJS/TEN who were admitted to King Fahad Specialist Hospital (KFSH) in Qassim region, Saudi Arabia, for the period between Jan 2014 to Jan 2019. The Careware information health system is used at KFSH, and patients were identified searching the diagnosis SJS/TEN.

**Results:**

Total of 10 patients with diagnosis of SJS/TEN were admitted to KFSH for the period from Jan 2014 to Jan 2019. Antibiotics were the culprit in 5 out of 10 patients. 9 out of 10 patients survived with good outcome. One patient with the diagnosis of TEN died, given extensive skin involvement complicated by sepsis.

**Conclusion:**

Despite the limitation of this study given small sample size, this is the first study of its kind that discusses the epidemiology of SJS/TEN in Saudi Arabia. We found the estimated incidence rate of SJS/TEN in Qassim region to be 7.6 cases per million person-years. Antibiotics and antiepileptics were the culprits in 8 out of 10 patients.

## 1. Introduction

Toxic epidermal necrolysis and Stevens-Johnson syndrome are acute inflammatory skin reactions [1]. SJS/TEN can affect any age group but is more prevalent in women, patients with HIV, and elderly. The global incidence rate of SJS/TEN is low [2].

Studies showed increased incidence with age and the number of medications taken [3]. The onset is generally caused by exposure to a medication such as nonsteroidal anti-inflammatory agents, antibiotics, and anticonvulsants [3]. It generally presents with skin blistering and desquamation with involvement of mucosal surfaces.

Toxic epidermal necrolysis is the most serious type of drug-induced skin reactions and involves >30% body surface area (BSA). Stevens-Johnson syndrome involves <10% of BSA while Stevens-Johnson syndrome/toxic epidermal necrolysis overlap involves 10%–30% of BSA. Mucous membranes such as oral, genital, anal, nasal, and conjunctival mucosa are typically involved in toxic epidermal necrolysis and Stevens-Johnson syndrome. Toxic epidermal necrolysis is associated with 30%–50% mortality and long-term sequelae [3]. Treatment involves early cessation of causative medication, supportive care, and admission to burn unit if needed [1]. Specific treatment with immunosuppressive drugs or immunoglobulins showed conflicting results regarding outcomes, and it remains controversial [1, 2]. The disease mechanism is not fully understood, but it appears that there are immunological mechanisms, cytotoxic reactions, and delayed hypersensitivity that could be involved in the pathogenesis [1, 2].

The rationale of this study is to explore the epidemiology and mortality rate of SJS/TEN in Saudi Arabia. There is no published data about SJS/TEN epidemiology in Saudi Arabia or other gulf countries, and despite the small sample size, we hope that this study will shed some light on such an important topic and hopefully will encourage other researchers in the region to conduct a larger, multicenter study for better understanding of this life-threatening condition.

## 2. Methodology

A retrospective cohort study was conducted at King Fahad Specialist Hospital (KFSH) in Qassim region, Saudi Arabia. KFSH is a tertiary hospital with a capacity of 420 beds, and it is the largest hospital in Qassim region which has a population of 1.3 million and located in the center of Saudi Arabia. Prior to starting the study, the ethical approval was obtained from Qassim Local Research Ethics committee (QREC). The hospital uses Careware information health system, and we identified all study patients by searching the diagnosis SJS, TEN, or SJS/TEN spectrum for the period from Jan 2014 to Jan 2019 from all hospital services. Ten patients were identified who met the diagnosis of SJS, TEN, or SJS/TEN. We reviewed the following variables for each patient; age, gender, diagnosis, and year of the diagnosis, causative agents, treatment, duration of hospital stay, and survival outcome.

## 3. Result

We identified 10 patients from KFSH hospital who met the diagnosis of SJS, TEN or SJS/TEN and were admitted between Jan 2014 to Jan 2019. Five patients were between 16 and 36 years old, three patients between 37 and 57 years old, and two patients were older than 57 years old. Six female patients and four were males.

Four patients were diagnosed with SJS, and six patients were diagnosed with TEN.

The causative agent of SJS/TEN was amoxicillin/clavulanic acid in 3 patients and carbamazepine in 2 patients. The culprit medications for the remaining 5 patients were levetiracetam, acetaminophen, azithromycin, ciprofloxacin, and unknown OTC medication in one patient. Four patients received IVIG plus supportive treatment, two patients were treated by IVIG in addition to oral corticosteroid and supportive treatment, two patients were managed by corticosteroid plus supportive treatment, and remaining two patients were managed by supportive treatment only.

One out of the ten patients died and the remaining patients survived with good outcome (Table 1).

## 4. Discussion

SJS/TEN is an acute life-threatening condition typically caused by drug hypersensitivity [3]. There is no prior study that discussed the epidemiology of TEN/SJS in the kingdom of Saudi Arabia or other gulf countries. We compared our results with another epidemiological study in the Middle East that was conducted in Israel [4]. They reviewed 26 patients with SJS/TEN, and they found a higher percentage of patients with SJS compared with our results. 65% of total study patients had SJS compared with 40% of total patients in our study. There was no significant difference in demographics between the 2 studies. The mortality rate was 15% compared with 10% in our study (one death). Antiepileptic drugs were the most common causative drugs, while antibiotics in our study were more common [4].

Epidemiology and incidence rate (IR) of SJS/TEN is a variable between different countries. A large observational study in the United Kingdom showed a TEN/SJS incidence ratio (IR) of 5.76 cases per million person-years in the UK [5]. Another epidemiological study in the United States showed IR of SJS/TEN of 12.7 cases per million person-years [6]. In our study, overall total number of person-years at risk in Qassim region was 1.3 million (Qassim population), and we estimated the IR to be 7.6 cases per million person-years.

The most commonly causative medications reported in the literature are antibiotics, anticonvulsants, allopurinol, and NSAID [3, 7, 8].

The gender distribution in our study was similar to published data in the literature [5, 9]. The mean age (SD) in this study sample was 38 ± (19.3). With regards to duration of hospital stay in this study, the average was 14.2 which is shorter than the average length of hospital stay reported by Chan et al. which was 20 days (range of 1–133 days) [10].

Among four patients in this study who received IVIG, one patient died which is comparable to the mortality rate reported in Israel 4 and was comparable to the mortality rate in Puerto Rico, USA, reported by Carrasquillo et al. who studied 30 patients with the diagnosis of SJS/TEN and found the mortality rate to be 12.5% [11].

The patient who died in our study was 35-year-old female, diagnosed with TEN with more than 90% skin involvement complicated by sepsis. There was a delay in her transfer from a nearby private hospital where she was managed by supportive care only with no other interventions.

Limitations of this retrospective study include small sample size and the fact that it is a single center study. We also could not estimate the relative exposure to the culprit medications in the population, and this can affect the relative risk of those culprit medications in causing SJS/TEN.

To explore the epidemiology of this life-threatening condition in more depth in Saudi Arabia, we recommend a larger study sample with participation of multiple centers in Saudi Arabia.

## 5. Conclusion

Despite the limitation of this study given small sample size, this is the first study of its kind that discusses the epidemiology of SJS/TEN in Saudi Arabia. We found the estimated incidence rate of SJS/TEN to be 7.6 cases per million person-years. Antibiotics and antiepileptics were the culprits in 8 out of 10 patients.

## Figures and Tables

**Table 1:** The demographics, clinical characteristics, and outcome of study patients (*N* = 10).

	Gender	Age	Diagnosis	Culprit medication	Comorbidities	Length of stay	Mortality outcome
Patient no. 1	Female	35	TEN	Amoxicillin/clavulanic acid	—	6 days	Died
Patient no. 2	Female	16	TEN	Amoxicillin/Clavulanic acid	—	70 days	Alive
Patient no. 3	Male	42	TEN	Amoxicillin/Clavulanic acid	Bronchial asthma	19 days	Alive
Patient no. 4	Female	43	SJS	Acetaminophen	—	7 days	Alive
Patient no. 5	Male	17	SJS	Unknown drug	—	3 days	Alive
Patient no. 6	Male	26	SJS	Azithromycin	—	4 days	Alive
Patient no. 7	Female	74	SJS	Ciprofloxacin	HTN, PE, CVA	5 days	Alive
Patient no. 8	Female	45	TEN	Levetiracetam	Epilepsy, HTN, hypothyroidism, CVA	6 days	Alive
Patient no. 9	Male	20	TEN	Carbamazepine	Epilepsy	14 days	Alive
Patient no. 10	Female	62	TEN	Carbamazepine	Bronchial asthma, HTN	8 days	Alive

## Data Availability

The patient data used to support the findings of this study are included within the supplementary information file. Clinical data and demographics are available within the article.

## Supplementary Materials

Supplementary Materials Supplementary data contains clinical and demographic details of each patient and it includes the admission dates.
