# Supplementary material for: Toxic Epidermal Necrolysis (TEN)/Stevens-Johnson Syndrome (SJS) Epidemiology and Mortality Rate at King Fahad Specialist Hospital (KFSH) in Qassim Region of Saudi Arabia: A Retrospective Study
Source: Dermatol Res Pract. 2020 Oct 9;2020:7524726. doi: 10.1155/2020/7524726 (PMC7568810; doi:10.1155/2020/7524726)
Supplement: Supplementary Materials — Supplementary data contains clinical and demographic details of each patient and it includes the admission dates. [file 7524726.f1.pdf]

(1)

Toxic Epidermal Necrolysis (TEN)/Steven Johnson Syndrome (SJS) Epidemiology and Mortality Rate in KFSH

Patient name	[redacted]				
File NO.	[redacted]				
Gender	MALE { }		FEMALE {✓}		
Diagnosis	SJS { }	SJS/TEN { }		TEN {✓}	
Year of Diagnosis (2017 / Q)	~~12~~ 3-3-1439 H	Age		35 yr.	
Causative agents	DRUG { } Amox		Other		
Management	supportive only				
Duration of stay in hospital	6 d				
Mortality outcome	☐ Alive ☑ Died				

because of sepsis

Extra Info			
Co - Morbidities	—	Readmission	
Treatment outcome	☐ Excellent ☐ Good ☐ Fair ☑ Poor		
Notes about the file	100% skin loss died of		
Data Entry Date	sepsis came late from		

Arabib hospital after about 10d

18-3 → admit

23-3- → discharge

Scanned with CamScanner

②

Toxic Epidermal Necrolysis (TEN)/Steven Johnson Syndrome (SJS) Epidemiology and Mortality Rate in KFSH

2016

Patient name	[redacted]		
File NO.	1435312		
Gender	MALE { }	FEMALE { 4	
Diagnosis	SJS { }	SJS/TEN { }	TEN { 4
Year of Diagnosis	21/6/37 H	Age	16
Causative agents	DRUG { } AMok/clav (augmintin)	Other	
Management	IVIG + Supportive plasma pheresis		
Duration of stay in hospital	20 d		
Mortality outcome	☑ Alive ☐ Died		

Extra Info				
Co - Morbidities	—	Readmission		
Treatment outcome	☐ Excellent	☑ Good	☐ Fair	☐ Poor
Notes about the file	complic: Symmetric priphral gangrin			
Data Entry Date	2/1/2020			

30/3/2016 admi

Scanned with CamScanner

(3)

Toxic Epidermal Necrolysis (TEN)/Steven Johnson Syndrome (SJS) Epidemiology and Mortality Rate in KFSH

Patient name	[redacted]			
File NO.	[redacted]			
Gender	MALE {✓}		FEMALE { }	
Diagnosis	SJS { }	SJS/TEN { }	TEN {✓}	
Year of Diagnosis	30/10/2015	Age	42 Y.O	
Causative agents	DRUG {✓} amoxicilin	Other		
Management	IV immunoglobulin + Supportive Care			
Duration of stay in hospital	19 days			
Mortality outcome	☑ Alive	☐ Died		

Extra Info				
Co - Morbidities	Bronchial A.	Readmission	NO only once	
Treatment outcome	☑ Excellent	☐ Good	☐ Fair	☐ Poor
Notes about the file				
Data Entry Date	2/1/2020			

ad: 17/1/1437
5/2/1437

Scanned with CamScanner

LX

Toxic Epidermal Necrolysis (TEN)/Steven Johnson Syndrome (SJS) Epidemiology and Mortality Rate in KFSH

Patient name	[redacted]		
File NO.	[redacted]		
Gender	MALE { }	FEMALE {✓}	
Diagnosis	SJS {✓}	SJS/TEN { }	TEN { }
Year of Diagnosis	15/1/2015	Age	43 Y.O
Causative agents	DRUG {✓} paracetamol	Other food (chees)	
Management	IVIG + supportive care + oral prednisolone (CS)		
Duration of stay in hospital	7 days		
Mortality outcome	☑ Alive ☐ Died		

Extra Info			
Co - Morbidities	—	Readmission	—
Treatment outcome	☑ Excellent ☐ Good ☐ Fair ☐ Poor		
Notes about the file			
Data Entry Date	2/1/2020		

24 3 36
Dis 29/3/36

Scanned with CamScanner

Toxic Epidermal Necrolysis (TEN)/Steven Johnson Syndrome (SJS) Epidemiology and Mortality Rate in KFSH

5

Patient name	[redacted]		
File NO.	[redacted]		
Gender	MALE {✓}	FEMALE { }	
Diagnosis	SJS {✓}	SJS/TEN { }	TEN { }
Year of Diagnosis	13/10/2015	Age	17
Causative agents	DRUG {✓} ....unknown..........	Other	
Management	- oral prednosolone + supportive care		
Duration of stay in hospital	3 days		
Mortality outcome	☑ Alive ☐ Died		

Extra Info			
Co - Morbidities	—	Readmission	—
Treatment outcome	☑ Excellent ☐ Good ☐ Fair ☐ Poor		
Notes about the file			
Data Entry Date	2/1/2020		

29/12
Dis 2/1/37

Scanned with CamScanner

0

Toxic Epidermal Necrolysis (TEN)/Steven Johnson Syndrome (SJS) Epidemiology and Mortality Rate in KFSH

2017

Patient name	[redacted]			
File NO.	[redacted]			
Gender	MALE { 4		FEMALE { }	
Diagnosis	SJS { 4	SJS/TEN { }	TEN { }	
Year of Diagnosis	9/6/38H	Age	26	
Causative agents	DRUG { } azithromycin	Other		
Management	oval pridisinolon + supportive			
Duration of stay in hospital	4 d			
Mortality outcome	☑ Alive	☐ Died		

	Extra Info			
Co - Morbidities	—	Readmission		
Treatment outcome	☑ Excellent	☐ Good	☐ Fair	☐ Poor
Notes about the file				
Data Entry Date	2-1-2020			

Scanned with CamScanner

7

Toxic Epidermal Necrolysis (TEN)/Steven Johnson Syndrome (SJS) Epidemiology and Mortality Rate in KFSH

Patient name	[redacted]		
File NO.	[redacted]		
Gender	MALE { }	FEMALE { ✓	
Diagnosis	SJS { ✓	SJS/TEN { }	TEN { }
2018 ← Year of Diagnosis	20/12/~~1~~ 39 H	Age	74 Y
Causative agents	DRUG { ✓ ciprofloxacin	Other	
Management	· supportive care		
Duration of stay in hospital	5 days		
Mortality outcome	☑ Alive ☐ Died		

Extra Info				
Co - Morbidities	HTN, PE, CVA	Readmission	X	
Treatment outcome	☑ Excellent	☐ Good	☐ Fair	☐ Poor
Notes about the file				
Data Entry Date	2/1/2020			

Scanned with CamScanner

8

Toxic Epidermal Necrolysis (TEN)/Steven Johnson Syndrome (SJS) Epidemiology and Mortality Rate in KFSH

Patient name	[redacted]			
File NO.	1328294			
Gender	MALE { }		FEMALE {✓	
Diagnosis	SJS { }	SJS/TEN { }	TEN {✓	
Year of Diagnosis (2019)	16-7-440H	Age	45Y	
Causative agents	DRUG { } levetriacetam		Other	
Management	IVIG + oral steroid and supportive			
Duration of stay in hospital	6d			
Mortality outcome	☑ Alive	☐ Died		

Extra Info cornary heart disease				
Co - Morbidities	Epilipsy CVA HTN hypothyroid	Readmission		
Treatment outcome	☑ Excellent	☐ Good	☐ Fair	☐ Poor
Notes about the file				
Data Entry Date				

16-7-40H

21-7-40 H

Scanned with CamScanner

9

Toxic Epidermal Necrolysis (TEN)/Steven Johnson Syndrome (SJS) Epidemiology and Mortality Rate in KFSH

Patient name	[redacted]			
File NO.	[redacted]			
Gender	MALE { ✓ }		FEMALE { }	
Diagnosis	SJS { }	SJS/TEN { }	TEN { ✓ }	
Year of Diagnosis	30/12/2014	Age	20	
Causative agents	DRUG { ✓ } Carbamezepen (tegretol)	Other		
Management	IVIG + supportiv care (tobical skin care, nutritinal care & prevention of 2ry infection + eye care) by Abx			
Duration of stay in hospital	14 day			
Mortality outcome	☑ Alive	☐ Died		

Extra Info				
Co - Morbidities	epelipsy	Readmission		
Treatment outcome	☑ Excellent	☐ Good	☐ Fair	☐ Poor
Notes about the file				
Data Entry Date	2/1/2020			

ad 30/12
Dis

8/3/1436
22/3/ 36

Scanned with CamScanner

(10)

Toxic Epidermal Necrolysis (TEN)/Steven Johnson Syndrome (SJS) Epidemiology and Mortality Rate in KFSH

Patient name	[redacted]			
File NO.	[redacted]			
Gender	MALE { }		FEMALE {✓}	
Diagnosis	SJS { }	SJS/TEN { }	TEN {✓}	
Year of Diagnosis	29/5/2018	Age	62 Y.O	
Causative agents	DRUG {✓} Carbamazipen (tegretol)	Other	-	-
Management	IVIG + supportive care			
Duration of stay in hospital	8 days			
Mortality outcome	☑ Alive	☐ Died		

	Extra Info			
Co - Morbidities	BA, HTN	Readmission	—	
Treatment outcome	☑ Excellent	☐ Good	☐ Fair	☐ Poor
Notes about the file				
Data Entry Date	2/1/2020			

ad 14/9/39
22/9/39

Scanned with CamScanner
